# Nitrooleic Acid Attenuates Lipid Metabolic Disorders and Liver Steatosis in DOCA-Salt Hypertensive Mice

**DOI:** 10.1155/2015/480348

**Published:** 2015-03-11

**Authors:** Haiping Wang, Jing Sun, Zhanjun Jia, Tianxin Yang, Liang Xu, Bing Zhao, Kezhou Yu, Rong Wang

**Affiliations:** ^1^Department of Nephrology, Provincial Hospital Affiliated to Shandong University, No. 324 Jingwu Road, Jinan, Shandong 250013, China; ^2^Department of Internal Medicine, University of Utah and Salt Lake Veterans Affairs Medical Center, Salt Lake City, UT 84112, USA

## Abstract

Nitrooleic acid (OA-NO_2_) is endogenous ligands for peroxisome proliferator-activated receptors. The present study was aimed at investigating the beneficial effects of OA-NO_2_ on the lipid metabolism and liver steatosis in deoxycorticosterone acetate- (DOCA-) salt induced hypertensive mice model. Male C57BL/6 mice were divided to receive DOCA-salt plus OA-NO_2_ or DOCA-salt plus vehicle and another group received neither DOCA-salt nor OA-NO_2_ (control group). After 3-week treatment with DOCA-salt plus 1% sodium chloride in drinking fluid, the hypertension was noted; however, OA-NO_2_ had no effect on the hypertension. In DOCA-salt treated mice, the plasma triglyceride and total cholesterol levels were significantly increased compared to control mice, and pretreatment with OA-NO_2_ significantly reduced these parameters. Further, the histopathology of liver exhibited more lipid distribution together with more serious micro- and macrovesicular steatosis after DOCA-salt treatment and that was consistent with liver tissue triglyceride and nonesterified fatty acids (NEFA) content. The mice pretreated with OA-NO_2_ showed reduced liver damage accompanied with low liver lipid content. Moreover, the liver TBARS, together with the expressions of gp91phox and p47phox, were parallelly decreased. These findings indicated that OA-NO_2_ had the protective effect on liver injury against DOCA-salt administration and the beneficial effect could be attributed to its antihyperlipidemic activities.

## 1. Introduction 

Hypertension is the most common cardiovascular disease and the prevalence of hypertension is projected to increase globally, especially in the developing countries. The metabolic abnormalities including lipid metabolism in hypertensive patients draw attention of the researchers considering the close association between them. There is increasing evidence for the strong relationship of hypertension and dyslipidemia. Metabolic abnormalities including hypertriglyceridemia, hypercholesterolemia, and insulin resistance were found in many hypertensive patients [1, 2]. Hypertension and dyslipidemia are major risk factors for cardiovascular disease, accounting for the highest morbidity and mortality among the patient population with cardiovascular disease. Hence, enormous studies have been focused on developing therapeutic agents for hypertension and its related lipid metabolism. There are several classical animal models existing for the hypertension study. Deoxycorticosterone acetate- (DOCA-) salt induced hypertensive animal model has been widely used for studying hypertension and hypertension-related organ damage. In the past decades, the increase of both circulating and membrane lipids was observed in DOCA-salt hypertensive rats prior to induction of hypertension [3, 4]. Meanwhile, the alteration of membrane phospholipids content, phospholipid distribution, and degree of fatty acid saturation were found in DOCA-salt hypertensive animals [3]. The changes in membrane lipid composition may modulate membrane function in a long-term mode, contributing to blood pressure regulation and heart, liver, and kidney organ dysfunction.

Nitrated free fatty acids (NO_2_-FA), notably nitroalkene derivatives of linoleic acid (nitrolinoleic acid) and nitrooleic acid (OA-NO_2_), are endogenous molecules with several attractive signaling properties [5, 6]. Nitroalkenes are found to be robust endogenous ligands for peroxisome proliferator-activated receptor-*γ* (PPAR*γ*), although they also activate PPAR*α* and PPAR*δ* at increasing concentrations [5, 6]. The agonists of PPARs are therapeutically used to treat dyslipidemia and hyperglycaemia associated with the metabolic syndrome. However, the serious side effects of certain PPARs agonists such as PPAR*γ* agonists from the thiazolidinedione type led to its limitation in clinical practice, which further increased the demand for the discovery of novel ligands. The endogenous ligand for PPARs has proven historically to be a promising pool of structures for drug discovery, and a significant research effort has recently been undertaken to explore the biological functions of the natural products. In a previous study, it was demonstrated that OA-NO_2_ significantly reduced the plasma triglycerides, almost normalized the plasma free fatty acids, and prevented the body weight gain without fluid retention in obese Zucker rats [7]. The present study aimed to examine the potential therapeutic effects of OA-NO_2_ for the dyslipidemia in DOCA-salt induced hypertensive mice.

## 2. Materials and Methods

### 2.1. Animals

Male C57BL/6 mice (8–10-week-old) were purchased from the Animal Center of Shandong University. All animals were fed using standard rodent chow with hand free access to water, and 12-hour light/dark cycle was maintained. All protocols employing mice were conducted in accordance with the principles and guidance of the Ethics Committee of the Provincial Hospital Affiliated to Shandong University.

### 2.2. Materials

9- and 10-nitrooleic acids are two regioisomers of OA-NO_2_, which are formed by nitration of oleic acid in approximately equal proportions in vivo [8]. Both compounds were purchased from Cayman Chemical (Ann Arbor, MI, USA), dissolved in dimethyl sulfoxide (DMSO), and used as a 1 : 1 mixture of the isomers.

### 2.3. Animal Experiments

The OA-NO_2_ was dissolved in 100% DMSO at 100 mg/mL. The mice were randomly divided into the following groups, mice were pretreated for 48 h with DMSO (DOCA-salt + vehicle) or OA-NO_2_ (DOCA-salt + OA-NO_2_) at 2 mg/kg/d via a microosmotic pump (DURECT Corporation, Cupertino, CA, USA), and then both groups were subject to DOCA-salt treatment. Under anesthesia with isoflurane, a slow-release (21-day) 50 mg DOCA pellet was implanted subcutaneously through a midscapular incision. Sham-operated animals served as controls. After the surgery, animals received 1% sodium chloride in drinking water and a normal salt diet for 3 weeks. Food intake, water intake, body weight, and urine volume were determined once per week in metabolic cages. At the end of the experiments, animals were fasted overnight before blood sampling, which was performed by making a small cut (~2 mm) in the tail using razor blade.

### 2.4. Blood Pressure Measurement

Systolic blood pressure was measured by a tail-cuff method using a Visitech BP2000 Blood Pressure Analysis System (Apex, NC, USA). All animals were habituated to the blood pressure measurement device for 7 days. All mice underwent 2 cycles of 20 measurements reordered per day for a minimum of 3 days.

### 2.5. Measurement of Biochemical Parameters and Cytokine

Blood samples from anesthetized mice were collected by puncturing vena cava using 1 cc insulin syringe containing 50 *μ*L of 1 mM ethylenediaminetetraacetic acid in the absence of protease inhibitors. Plasma levels of triglyceride, cholesterol, aspartate aminotransferase (AST), and alanine aminotransferase (ALT) were measured using a blood chemistry analyzer.

For analysis of hepatic triglyceride and nonesterified fatty acids (NEFA), liver tissues were ground in liquid nitrogen and dissolved in 0.9% sodium chloride. The NEFA levels were determined from the supernatant using the commercial enzymatic colorimetric kits (Wako Chemicals, VA, USA), according to the manufacturer's instructions. The liver triglyceride levels were determined using an L-type triglyceride H kit (Wako Chemicals, VA, USA), according to the manufacturer's instructions.

### 2.6. Histopathological Analysis

After 3 weeks of treatment with DOCA-salt, the mice were euthanized and the livers were excised, fixed in 4% paraformaldehyde, and embedded in paraffin. The paraffin embedded tissues were sectioned at 4 *μ*m and stained with hematoxylin and eosin (HE) by standard methods. Hepatic steatosis was blindly assessed on 4 random fragments from different areas of each liver and was staged on a scale of 0 to 4, according to the percentage of hepatocytes containing cytoplasmic vacuoles as follows: 0 (<5%), 1 (5–20%), 2 (20%–30%), 3 (30–60%), and 4 (≥60%). Frozen samples were also stained with Oil-Red-O at the optimal cutting temperature. Slides were analyzed by microscopy using Image J and Microsuite (Olympus Soft Imaging Solutions GmbH, Munster, Germany).

### 2.7. Measurement of Thiobarbituric Acid-Reactive Substances

The measurement of plasma thiobarbituric acid-reactive substances (TBARS) was based on the formation of malondialdehyde using a commercially available TBARS Assay Kit (10009055; Cayman Chemical, Ann Arbor, MI, USA), according to the manufacturer's instructions.

### 2.8. Real-Time Polymerase Chain Reaction (RT-PCR)

Mice livers were harvested and preserved in the RNAlater solution (Sangon Biotech, China) at −20°C until ribonucleic acid (RNA) extraction. Total RNA was isolated using TRIzol reagent (Invitrogen, CA, USA), and complimentary deoxyribonucleic acid was synthesized with SuperScript (TaKaRa Bio, Japan). RT-PCR was carried out using a QuantiTect SYBR Green Kit (Qiagen, Germany) on an ABI Prism 7500 RT-PCR instrument equipped with appropriate software (Applied Biosystems, CA, USA). The oligonucleotide sequences used for RT-PCR were as follows (Sangon Biotech, China): glyceraldehyde 3-phosphate dehydrogenase, sense 5′-GTCTTCACTACCATGGAGAGG-3′ and antisense 5′-TCATGGATGACCTTGGCCAG-3′; p47phox, sense 5′-GTCGTGGAGAAGAGCGAGAG-3′ and antisense 5′-CGCTTTGATGGTTACATACGG-3′; gp91phox, sense 5′-CCGTATTGTGGGAGACTGGA-3′.

### 2.9. Statistical Analysis

All values were presented as mean ± standard deviation. Statistical analysis was performed using a student* t-*test or analysis of variance. A value of *P* < 0.05 was considered statistically significant.

## 3. Results

### 3.1. Effect of OA-NO_2_ on Body Weight Loss

**Figure 1:**
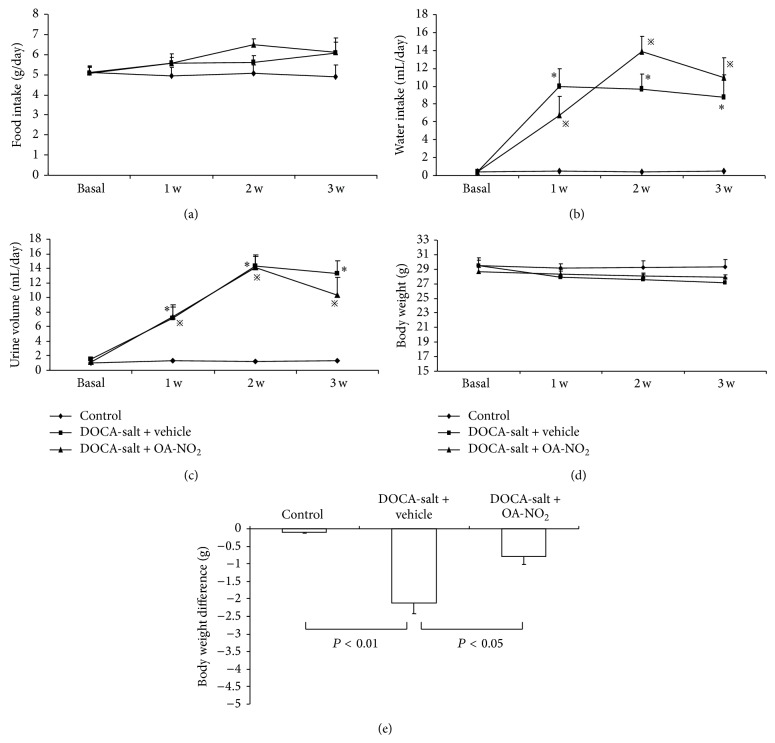
Food intake (a), water intake (b), urine volume (c), body weight (d), and body weight difference (e) in control (*n* = 5), DOCA-salt + vehicle (*n* = 8), and DOCA-salt + OA-NO_2_ (*n* = 8) mice. ^*^ *P* < 0.05, compared with control; ^*※*^ *P* < 0.05, compared with control; data are shown as mean + SE.

During the 3-week treatment, food intake, water intake, body weight, and urine volume were determined once per week in metabolic cages. There were no differences among the control, DOCA-salt + vehicle, and DOCA-salt + OA-NO_2_ groups in food intake (Figure 1(a)), while the water intake increased strikingly after implanting the DOCA pellet together with high salt drinking water; however, OA-NO_2_ has no effect on the alteration of the water intake (Figure 1(b)). The urine volume had the same pattern similar to water intake (Figure 1(c)). Although there were no differences in the food intake, the DOCA-salt treatment induced the body weight loss (control: −0.11 ± 0.02 versus DOCA-salt + vehicle: −2.1 ± 0.31, *P* < 0.01), which was less in OA-NO_2_ treatment group (DOCA-salt + OA-NO_2_: −0.79 ± 0.23) (*P* < 0.05) (Figure 1(e)).

### 3.2. Effect of OA-NO_2_ on Blood Pressure

**Figure 2:**
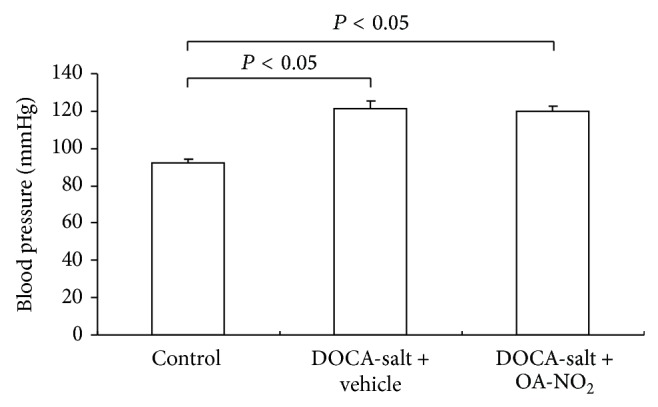
The changes of blood pressure in control (*n* = 5), DOCA-salt + vehicle (*n* = 8), and DOCA-salt + OA-NO_2_ (*n* = 8) mice after 3 weeks of DOCA-salt treatment. Data are shown as mean + SE.

After 3-week treatment with DOCA-salt, the mice exhibited higher blood pressure than control mice (control: 92.5 ± 2.23 versus DOCA-salt + vehicle: 121.5 ± 4.38, *P* < 0.05); however, OA-NO_2_ had no effect on the hypertension (Figure 2). The result was different from the previous study results where the OA-NO_2_ inhibited angiotensin II-induced hypertension [12].

### 3.3. Effect of OA-NO_2_ on Biochemical Parameters

**Figure 3:**
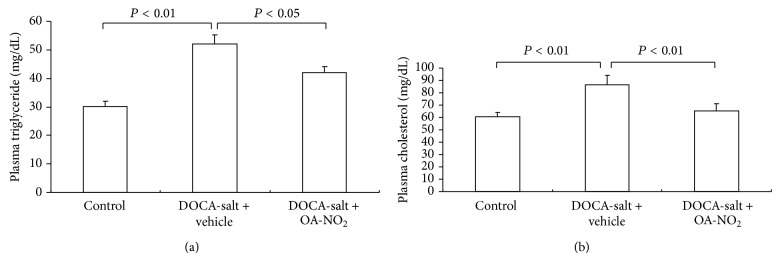
Effect of OA-NO_2_ on plasma triglyceride (a) and cholesterol (b) in DOCA-salt hypertensive mice. Control: *n* = 5; DOCA-salt + vehicle: *n* = 8; DOCA-salt + OA-NO_2_: *n* = 8. Data are shown as mean + SE.

After 3-week treatment, plasma triglyceride concentrations increased in DOCA-salt treated mice (control: 30.1 ± 2.02 versus DOCA-salt + vehicle: 51.9 ± 3.29, *P* < 0.01), which was reduced to 42.1 ± 2.19 by OA-NO_2_ treatment (*P* < 0.05) (Figure 3(a)). A similar increase in the plasma cholesterol concentration after the treatment with DOCA-salt (control: 60.3 ± 4.03 versus DOCA-salt + vehicle: 86.5 ± 7.78, *P* < 0.01) was observed, and the pretreatment with OA-NO_2_ almost normalized the plasma cholesterol levels (Figure 3(b)).

**Figure 4:**
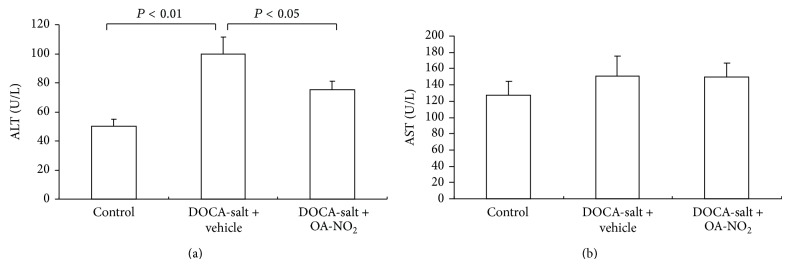
Effect of OA-NO_2_ on plasma AST and ALT after 3-week DOCA-salt treatment. Control: *n* = 5; DOCA-salt + vehicle: *n* = 8; DOCA-salt + OA-NO_2_: *n* = 8. Data are shown as mean + SE.

The liver is sensitive to the dyslipidemia, and DOCA-salt treated rats showed fatty changes in hepatocytes in the previous study [9]. Hence, the liver damage was assessed by ALT and AST levels. The plasma ALT rose significantly after DOCA-salt treatment compared with control mice (control: 50.3 ± 5.2 versus DOCA-salt + vehicle: 100.5 ± 11.3, *P* < 0.05), while the OA-NO_2_ treatment attenuated the liver injury with reduced ALT (75.5 ± 6.2, *P* < 0.05) (Figure 4(a)). The DOCA-salt treated mice had slight higher plasma AST values than the control mice; however, there were no statistical differences among the three groups (Figure 4(b)).

### 3.4. Effect of OA-NO_2_ on Liver Steatosis

**Figure 5:**
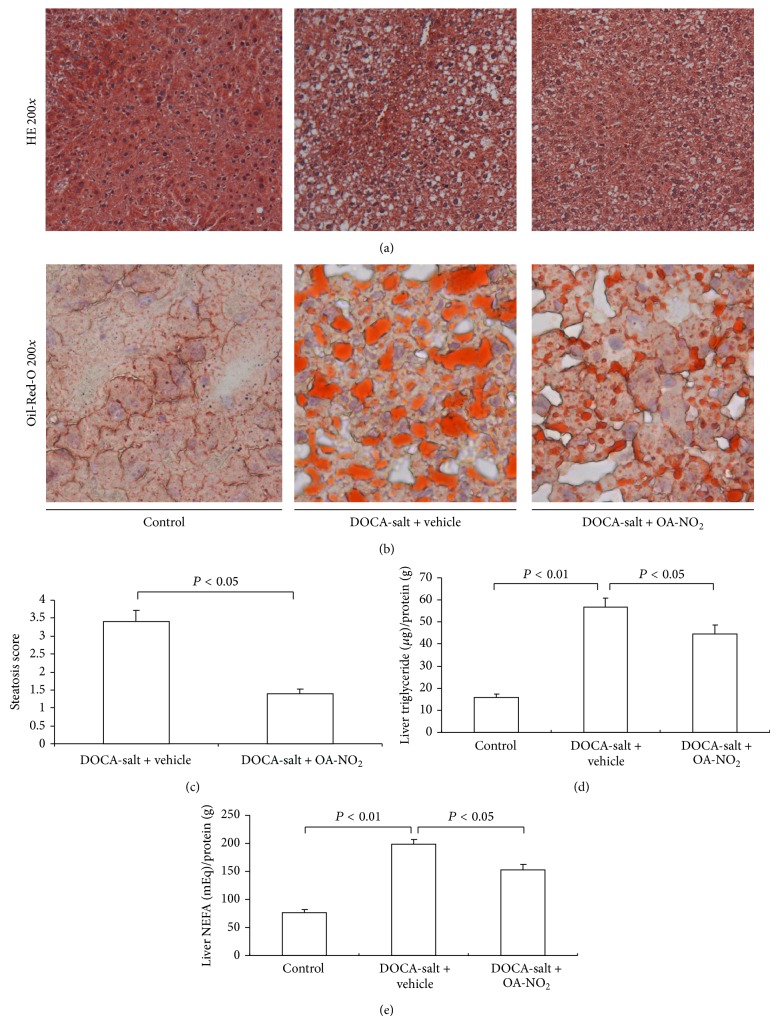
Effect of OA-NO_2_ on the liver steatosis after 3-week DOCA-salt treatment. Morphological analysis of DOCA-salt treated liver injury in control, DOCA-salt + vehicle, and DOCA-salt + OA-NO_2_ mice. (a) Representative photomicrographs: hematoxylin and eosin staining (magnification 200). (b) Oil-Red-O staining (magnification 200) of livers. (c) Mean liver steatosis score. The lipid content in liver: liver triglyceride (d) and liver NEFA (e). Control: *n* = 5; DOCA-salt + vehicle: *n* = 8; DOCA-salt + OA-NO_2_: *n* = 8. Data are shown as mean + SE.

To assess liver steatosis, the liver sections were evaluated histologically using HE and Oil-Red-O staining. The DOCA-salt treated mice showed significantly more micro- and macrovesicular steatosis, characterized with hypertrophy and globular hyalinization, edema, and hydrophobic changes than control mice (Figures 5(a) and 5(b)), and the pretreatment with OA-NO_2_ attenuated the histological changes in the liver, as illustrated by the decrease in the steatosis score (Figure 5(c)). With Oil-Red-O staining, the DOCA-salt treated mice showed more lipid accumulation in the liver than the control, while the OA-NO_2_ strikingly reduced the lipid accumulation.

After the histological staining, the fatty liver was further evaluated for triglyceride and NEFA levels in the liver tissue. After 4-week treatment, the DOCA-salt treated mice had significantly elevated liver triglyceride (control: 15.9 ± 1.45 versus DOCA-salt + vehicle: 56.7 ± 4.21, *P* < 0.01) and NEFA (control: 76.3 ± 5.80 versus DOCA-salt + vehicle: 199.1 ± 8.41, *P* < 0.01) levels compared to control mice (Figures 5(d) and 5(e)), while the OA-NO_2_ treated mice had less liver triglyceride (44.6 ± 3.91) and NEFA (153.2 ± 9.89) levels compared to DOCA-salt treated mice.

### 3.5. Effect of OA-NO_2_ on Oxidative Stress in Liver

**Figure 6:**
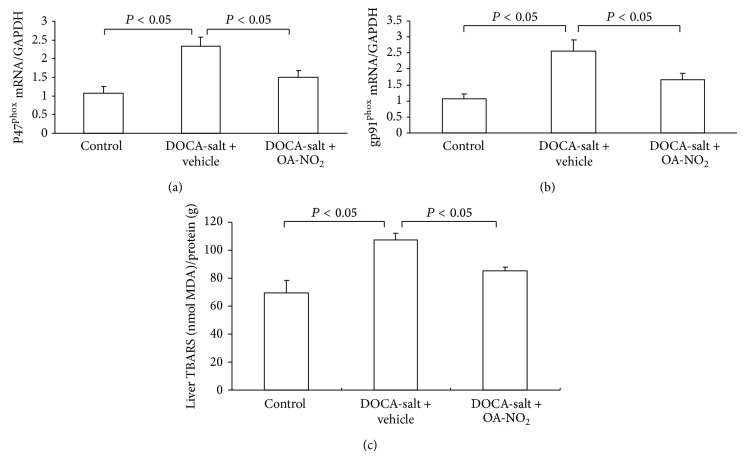
Effect of OA-NO_2_ on the oxidative stress after 3-week DOCA-salt treatment. Liver p47^phox^ mRNA expression (a) and gp91^phox^ mRNA expression, and the liver TBARS content (c) in control (*n* = 5), DOCA-salt + vehicle (*n* = 8), and DOCA-salt + OA-NO_2_ (*n* = 8) mice. Data are shown as mean + SE.

The presence of oxidative stress in the liver was evaluated. In the livers of DOCA-salt treated mice, TBAR levels showed 40% increase compared to the control mice; however, this increase was less (24%) in OA-NO_2_ treated mice. To understand the liver sources of oxidative stress and liver expressions of gp91phox and p47phox, the two major nicotinamide adenine dinucleotide phosphate (NADPH) oxidase subunits, along with superoxide dismutase- (SOD-) 1 and SOD2, were examined using quantitative RT-PCR. DOCA-salt treatment induced parallel increases in renal gp91phox (2.5-fold; *P* < 0.05) and p47phox (2.3-fold; *P* < 0.05) without any effect on SOD1 or SOD2 compared with control mice (data did not show), and these increases were significantly reduced by OA-NO_2_ treatment (Figure 6).

## 4. Discussion

Nitrated fatty acids are derived from NO- and NO_2_-dependent redox reactions with unsaturated fatty acids, including OA-NO_2_ and nitrolinoleic acid [10]. The OA-NO_2_, a nitrated fatty acid and an endogenous PPARs agonist, has been reported to have relevant biological effects on inflammation [11], hypertension [12], vascular neointimal proliferation [13], obesity with metabolic syndrome [7], hyperglycemia [14], and proteinuria [15] in diabetes without side effects. Hence, a significant research work has recently been undertaken to explore the biological functions of the natural products. In the present study, it was demonstrated that the pretreatment with OA-NO_2_ produced beneficial effects on hyperlipidemia and liver steatosis with no effect on hypertension in the DOCA-salt induced hypertensive mice model.

In the DOCA-salt induced hypertensive rat model, the animals showed significant elevation in mean arterial pressure, heart rate, and reduction in body weight. A significant increase in the plasma and tissue (liver, kidney, heart, and aorta) lipid levels such as total cholesterol, triglycerides, free fatty acids, and phospholipids was noted in DOCA-salt hypertensive rats [16, 17]. In the present study, it was found that the pretreatment with OA-NO_2_ lowered the plasma triglyceride and cholesterol concentrations, without any impact on the mice food intake. The present study results were different from previous data, which showed that OA-NO_2_ produced beneficial effects on obesity and hyperlipidemia, accompanied by an immediate reduction of food intake in obese Zucker rats [7]. The PPAR*α* agonists (Wy-14643 and GW7647) and endogenous PPAR*α* agonists (oleoylethanolamide) treatment reduced the body weight gain and improved the hyperlipidemia via appetite-suppressing effect (within a day) in an obese animal model wherein the hyperlipidemia was mainly due to the high food intake [18–21].

Unlike the obese Zucker rats, the mechanism of the hyperlipidemia of DOCA-salt hypertensive animals was different. Interestingly, in DOCA-salt hypertensive rats, Hernández et al. [22] found that the glucose, glycogen, and triglycerides levels were increased and citrate synthase and beta-hydroxyacyl-CoA dehydrogenase activities were reduced in the soleus muscle, along with higher plasma triglycerides and cholesterol compared with control rats; the study findings indicated that the hyperlipidemia of the DOCA-salt hypertensive animals was due to the changes of metabolic enzymes and not because of high food intake. The OA-NO_2_ not only is a robust PPAR*γ* activator but also activates PPAR*α* and PPAR*δ* at increasing concentrations. All three PPAR subtypes have been shown to play a crucial role in whole body lipid homeostasis as well as in insulin sensitivity [23–26]. In spite of the impact of PPAR*α* on the appetite-suppressing effect, both PPAR*γ* and PPAR*δ* have no effect on food intake [18, 27].

The mechanism of action of OA-NO_2_ in DOCA-salt hypertensive mice remains elusive; however, the involvement of PPARs appears conceivable. All three PPAR isoforms have been identified as therapeutic targets in the treatment of patients with hyperlipidaemia. Among these, PPAR*α* has been found to play a key role in lipid metabolism. PPAR*α*-null mice exhibited higher plasma levels of cholesterol and triglycerides [28] with extensive hepatic lipid accumulation. The PPAR*α* is known to be involved in almost all aspects of lipid metabolism including uptake, binding, and oxidation of fatty acids, lipoprotein assembly, and lipid transport [29]. The PPAR*γ* is also an important regulator in lipid homeostasis. The PPAR*γ* gene deficiency resulted in elevated plasma levels of triglycerides and NEFA [30]. The thiazolidinediones type PPAR*γ* activators including troglitazone and pioglitazone improved the dyslipidemia by lowering the triglyceride levels in patients with dyslipidemia [31, 32]. In line with these observations, PPAR*β*/*δ*-null mice on a high-fat diet showed an increased rate of hepatic very-low-density lipoprotein production, and treatment of various animal models with selective PPAR*β*/*δ* agonists (GW0742 and L165041) yielded valuable data favoring PPAR*β*/*δ* as a therapeutic target for dyslipidemia [33]. Based on the above-mentioned evidences, it can be concluded that all the three PPAR subtypes have the lipid lowering properties mediated via different signal pathways. It may be possible that the lipid lowering effects of OA-NO_2_ share the lipid lowering properties of three PPAR subtypes, without reducing the food intake of animals. There were also the possibilities that the OA-NO_2_ reduced the lipids via PPARs independent pathways.

Besides the hyperlipidemia, the fatty liver also was present in the DOCA-salt hypertensive mice. The DOCA-salt treated rats showed fatty changes in hepatocytes besides hypertrophy and globular hyalinization, edema, and hydrophobic changes [9]. In the present study, a similar liver steatosis characterized with striking histological changes along with abnormal ALT was found. Pretreatment of OA-NO_2_ strikingly attenuated the liver injury and reduced the liver tissue triglycerides. The DOCA-salt induced hypertension status is known to be associated with oxidative stress resulting from an imbalance of antioxidant defense mechanisms in various tissues, including an early imbalance of liver antioxidant. Hepatic antioxidant defenses were decreased as early as 1 week of hypertensive treatment; the decrease of peroxidase, reductase, transferase, and catalase activities was associated with a significant increase of TBARS levels [34]. On the other hand, the high triglyceride levels can trigger liver oxidative stress after a long-term high-fat diet [35]. Generally, lipid and metabolic disorders are very likely to cause oxidant-antioxidant imbalances in the liver, where high levels of fatty acids provide the material basis for oxidative stress [36, 37]. Excessive levels of FFAs induced high levels of *β*-oxidation, and the production of ROS decreased antioxidant defenses at a mitochondrial respiratory chain level, simultaneously with the induction of steatosis [38]. Expressions of p47phox and gp91phox have been found to increase in obese rats and to be partly responsible for excessive oxidation [39]. Considering the hyperlipidemia and fatty liver in DOCA-salt induced hypertensive mice, the liver oxidative stress was accessed by examining liver TBARS levels and the two major NADPH oxidase subunits gp91phox and p47phox. The DOCA-salt hypertensive mice exhibited extensive liver oxidative stress, while OA-NO_2_ treatment improved the oxidative stress. The antioxidative stress effect of OA-NO_2_ is consistent with the previous study results in ischemia and reperfusion and endotoxemia mice model [11, 40].

In addition to the effect of OA-NO_2_ on obesity and obesity-related conditions [7], in the present study, it was demonstrated that OA-NO_2_ had the beneficial effect on the hypertension-related lipid metabolism. Hypertension is recognized globally as a major risk factor for cardiovascular disease, stroke, diabetes, and renal diseases [41]. There is a strong association between hypertension and dyslipidemia. About 80% of hypertensive patients have comorbidities such as obesity, glucose intolerance, and lipid metabolism abnormalities. Several prospective studies have found that the hypertensive patients had higher serum total cholesterol and triglycerides compared to healthy normotensive controls [42]. It is meaningful that the OA-NO_2_ has the potential properties for improving the hypertension-related lipid metabolism and organ injury.

In summary, OA-NO_2_ is a newly identified endogenous product with potent antioxidant and anti-inflammatory properties and has demonstrated a favorable safety profile in animal studies. The present study results demonstrated that the OA-NO_2_ restored the lipid metabolism and ameliorated the liver steatosis in DOCA-salt hypertensive mice. The results indicated the novel therapeutic potential of OA-NO_2_ in a rodent model of hypertension-related lipid metabolism.
